# Spatiotemporal Recurrent Convolutional Networks for Traffic Prediction in Transportation Networks

**DOI:** 10.3390/s17071501

**Published:** 2017-06-26

**Authors:** Haiyang Yu, Zhihai Wu, Shuqin Wang, Yunpeng Wang, Xiaolei Ma

**Affiliations:** 1 School of Transportation Science and Engineering, Beijing Key Laboratory for Cooperative Vehicle Infrastructure System and Safety Control, Beihang University, Beijing 100191, China; hyyu@buaa.edu.cn (H.Y.); zhihaiwu@buaa.edu.cn (Z.W.); ypwang@buaa.edu.cn (Y.W.); 2 Passenger Vehicle EE Development Department, China FAW R&D Center, Changchun 130011, China; wangshuqin@rdc.faw.com.cn

**Keywords:** traffic prediction, convolutional neural network, long short-term memory, spatiotemporal feature, network representation

## Abstract

Predicting large-scale transportation network traffic has become an important and challenging topic in recent decades. Inspired by the domain knowledge of motion prediction, in which the future motion of an object can be predicted based on previous scenes, we propose a network grid representation method that can retain the fine-scale structure of a transportation network. Network-wide traffic speeds are converted into a series of static images and input into a novel deep architecture, namely, spatiotemporal recurrent convolutional networks (SRCNs), for traffic forecasting. The proposed SRCNs inherit the advantages of deep convolutional neural networks (DCNNs) and long short-term memory (LSTM) neural networks. The spatial dependencies of network-wide traffic can be captured by DCNNs, and the temporal dynamics can be learned by LSTMs. An experiment on a Beijing transportation network with 278 links demonstrates that SRCNs outperform other deep learning-based algorithms in both short-term and long-term traffic prediction.

## 1. Introduction

Predicting large-scale network-wide traffic is a vital and challenging topic for transportation researchers. Traditional traffic prediction studies either relied on theoretical mathematical models to describe the traffic flow properties (i.e., model-driven approaches) or employed a variety of statistical learning and artificial intelligence algorithms (i.e., data-driven approaches). Model-driven approaches are criticized for strong assumptions and thus are inappropriate for application to real scenarios. Data-driven approaches have become increasingly popular due to the extensive deployment of traffic sensors and advanced data processing technologies. However, the majority of existing approaches tend to design and validate the proposed algorithms on expressways or several intersections [1,2]. As discussed in [3], most traffic prediction methods that consider both spatial correlations and temporal correlations limit the input dimensionality (i.e., the number of nearby road segments that contribute to the prediction) to 100. From the perspective of spatial analysis, traffic congestion that occurs at a single location may propagate to other regions that are located a significant distance away from the congested site. This phenomenon has been witnessed and confirmed by [3,4]. From the perspective of temporal analysis, a strong correlation usually exists among traffic time series, where previous traffic conditions likely have a large impact on future traffic. For a large-sized network with hundreds of links, capturing the spatial and temporal features of any link is very challenging [4,5]. Traditional model-based approaches rely on either traffic flow theory or a complex network to mimic the traffic congestion evolution, and typically involve a number of unrealistic assumptions such as time-invariant travel costs and homogeneous traveler route choices. Due to the emergence of big data and deep learning, predicting large-scale network traffic has become feasible due to abundant traffic sensor data and hierarchical representations in deep architectures.

In the domain of computer vision, deep learning has achieved a better performance than traditional image-processing paradigms. Deep learning in motion prediction is a research area in which the future movement of an object is predicted based on a series of historical scenes of the same object. Based on the success of this method, we snapshot network-wide traffic speeds as a collection of static images via a grid-based segmentation method, where each pixel represents the traffic condition of a single road segment or multiple road segments. As time evolves, the network-wide traffic prediction problem becomes a motion-prediction issue. Given a sequence of static images that comprise an animation, can we predict the future motion of each pixel? The deep-learning framework presents superior advantages in enhancing the motion prediction accuracy [6,7]. Both spatial and temporal long-range dependencies should be considered when a video sequence is learned. Convolutional neural networks (CNNs) adopt layers with convolution filters to extract local features through sliding windows [8] and can model nearby or citywide spatial dependencies [9]. To learn time series with long time spans, long short-term memory (LSTM) neural networks (NNs), which were proposed by Hochreiter and Schmidhuber [10] in 1997, have been effectively applied in short-term traffic prediction [11,12] and achieve a remarkable performance in capturing the long-term temporal dependency of traffic flow. Motivated by the success of CNNs and LSTMs, this paper proposes a spatiotemporal image-based approach to predict the network-wide traffic state using spatiotemporal recurrent convolutional networks (SRCNs). Deep convolutional neural networks (DCNNs) are utilized to mine the space features among all links in an entire traffic network, whereas LSTMs are employed to learn the temporal features of traffic congestion evolution. We input the spatiotemporal features into a fully connected layer to learn the traffic speed pattern of each link in a large-scale traffic network and train the model from end to end.

The contributions of the paper can be summarized as follows:

- We developed a hybrid model named the SRCN that combines DCNNs and LSTMs to forecast network-wide traffic speeds.
- We proposed a novel traffic network representation method, which can retain the structure of the transport network at a fine scale.
- The special-temporal features of network traffic are modeled as a video, where each traffic condition is treated as one frame of the video. In the proposed SRCN architecture, the DCNNs capture the near- and far-side spatial dependencies from the perspective of the network, whereas the LSTMs learn the long-term temporal dependency. By the integration of DCNNs and LSTMs, we analyze the spatiotemporal network-wide traffic data.

The remainder of this paper is organized as follows: Section 2 discusses the existing literature on traffic prediction. Section 3 introduces a grid-based transportation network representation approach for converting historical network traffic into a series of images and proposes the architecture of SRCNs to capture the spatiotemporal traffic features. In Section 4, a transportation network in Beijing with 278 links is employed to test the effectiveness of the proposed method. To evaluate the performance of SRCNs, we compare three prevailing deep learning architectures (i.e., LSTMs; DCNNs; and stacked auto encoders, SAEs) and a classical machine learning method (support vector machine, SVM). At the end of this paper, the conclusions are presented and future studies are discussed.

## 2. Literature Review

Short-term traffic forecasting has attracted numerous researchers worldwide and can be traced to the 1970s. The approaches can be divided into two groups: parametric approaches and nonparametric approaches [13].

### 2.1. Parametric Approaches

Parametric methods include the autoregressive integrated moving average (ARIMA), the Kalman filter (KF), and exponential smoothing (ES). Hamed et al. developed a simple ARIMA model of the order (0, 1, 1) to forecast the traffic volume on urban arterials [14]. Ding et al. classified the traffic modes into six classes and proposed a space-time autoregressive integrated moving average (STARIMA) model to forecast the traffic volume in urban areas in five-minute intervals [15]. S.R. Chandra and H. Al-Deek proposed vector autoregressive models for short-term traffic prediction on freeways that consider upstream and downstream location information and yield a high accuracy [16]. Motivated by the superior capability to cast the regression problem of a KF, numerous KF-based traffic prediction studies began to emerge [17,18,19]. S.H. Hosseini et al. applied an adaptive neuro fuzzy inference system (ANFIS) based on KF to address the nonlinear problem of traffic speed forecasting [20]. B. Williams et al. developed a traffic flow prediction approach based on exponential smoothing, and K.Y. Chan employed a smoothing technique to pre-process traffic data before inputting the data into NNs for prediction, which achieved more than a 6% accuracy [21,22].

### 2.2. Nonparametric Approaches

Compared with parametric approaches, nonparametric models are flexible and complex since their structure and parameters are not fixed. In the domain of nonparametric approaches, an SVM that is based on statistical learning theory is popular in the field of prediction [23]. The premise of an SVM is to map low-dimensional nonlinear data into a high-dimensional space by a kernel function. However, an SVM is highly sensitive to the choices of the kernel function and parameters. Many researchers have attempted to optimize an SVM and apply it to traffic prediction to derive some improved SVM variants, such as chaos wavelet analysis SVMs [24], least squares SVMs [25], particle swarm optimization SVMs [26], and genetic algorithm SVMs [27].

Another typical nonparametric method is an NN, which is extensively applied in almost every field, including that of traffic prediction. An NN can model complex nonlinear problems with a remarkable performance in handling multi-dimensional data [28]. S.H. Huang et al. constructed an NN model to predict traffic speed that considers weather conditions [29]. A. Khotanzad and N. Sadek applied a multilayer perceptron (MLP) and a fuzzy neural network (FNN) to high-speed network traffic prediction; the results indicate that NN performs better than the autoregressive model [30]. C. Qiu et al. developed a Bayesian-regularized NN to forecast short-term traffic speeds [31]. X. Ma [11] proposed a congestion prediction method that is based on recurrent neural networks and restricted Boltzmann machines (RNN-RBM) for a large-scale transportation network that included 515 road links.

**Figure 1:**
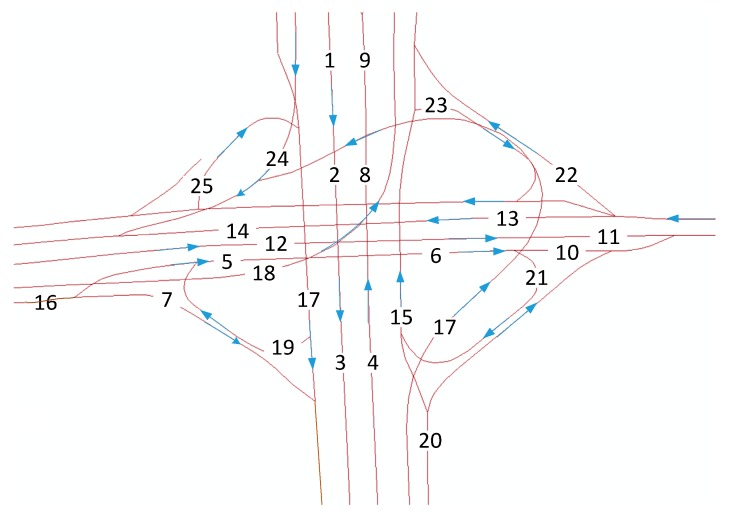
Layout of an interchange in Beijing, China.

In recent years, deep NNs, such as deep belief networks (DBNs), have been investigated in traffic flow prediction [32,33,34,35]. Although these methods are suitable for small-scale traffic networks or networks with few links, they fail to take advantage of correlations among different links and the long-term memory of traffic. To overcome these drawbacks, a special recurrent NN named LSTM is proposed to forecast the traffic speed and traffic flow (X. Ma [36], Y. Tian [37], Y. Chen [38], R. Fu [39]). The results indicate that LSTMs outperform MLP and SVMs. The temporal features of traffic can be mined by time-series algorithms, such as LSTMs; however, these algorithms always fail to capture the spatial features among links. The capability of CNNs to extract the spatial features in a local or city-wide region has been proven. Wu and Tan [40] constructed a short-term traffic flow prediction method based on the combination of CNNs and LSTMs on an arterial road. In this approach, the road is divided into several links to view the road as a vector. This vector is input into one-dimensional CNNs to capture the spatial features of the links, and LSTMs are utilized to mine the temporal characteristics. This method can extract spatiotemporal correlations on a single arterial road, but fails to consider ramps, interchanges, and intersections, which are significant components of any transportation network [41]. Consequently, the method disregards the spatial-propagation effect of congestion: a traffic incident that occurs on one link may influence the traffic conditions in far-side regions. Considering Figure 1 as an example, the four-way intersection with four ramps has 25 links. If an incident occurs on link 9, then link 8, link 15, and link 21 are very likely to be congested. One-dimensional CNNs cannot adequately capture the spatial relations among links 8, 9, 15, and 21 because the convolutional filter of one-dimensional CNNs can only include a finite number of consecutive traffic speeds along each link and is unable to consider the zonal spatial dependencies among links that are not adjacent to each other, such as link 16 and link 3. In this circumstance, a two-dimensional (2D) convolutional filter must be employed to address regional traffic conditions. This improvement is especially important for predicting traffic at interchanges and intersections.

To address these drawbacks, based on the traffic network level, this paper proposes a novel NN structure that combines deep 2D CNNs and deep LSTMs to obtain the spatiotemporal correlations among all links in a traffic network. Several successful applications have verified the feasibility of combining CNNs and LSTMs, such as image descriptions [42], visual activity recognition [43], and sentiment analysis [44]. Thus, we view the traffic network evolution process as a video, where every frame represents a traffic state and several future frames can be forecasted based on several previous frames. Based on this idea, the future traffic state can be effectively forecasted using well-established image-processing algorithms.

## 3. Methodology

In this section, we construct our SRCNs for predicting the traffic state. An SRCN consists of a 2D CNN and two LSTMs; the details are presented in the following section.

### 3.1. Network Representation

Assume that we want to predict the congestion at every link in a traffic network. We establish the links ${\left\{y_{i}\right\}}_{i=1}^{n}$ , where n represents the total number of links.

**Figure 2:**
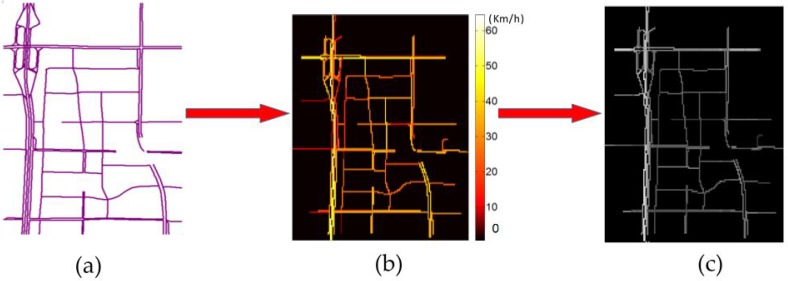
Grid-based transportation network segmentation process: (**a**) A transportation network without traffic information; (**b**) the speed of each link is mapped and colored in the network; (**c**) the speed of each link is normalized and greyed in the network.

**Figure 3:**
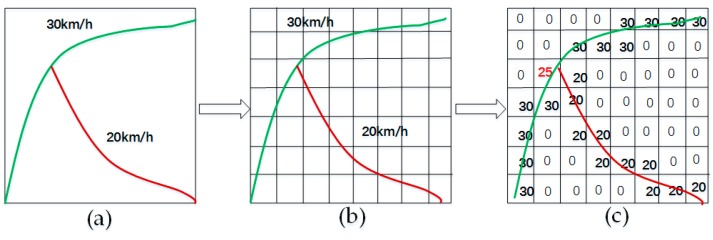
Traffic speed representation in a small-scale transportation network: (**a**) two links with different speeds in a transportation network; (**b**) the network is divided into several grids (the size of each grid is approximately 10 m × 10 m); (**c**) the speed of each link is mapped to each grid.

Step 1: We choose a traffic network (refer to Figure 2a), divide it into n links according to the road condition, and calculate the average speeds on these links over a particular time period, which is set to two minutes according to Equation (1), where m and ${\bar{v}}_{j}$ represent the number of vehicles and their average speed, respectively, on link j. We map the calculated speeds on the links using different colors (as shown in Figure 2b). Figure 3a shows an example of a small-scale network.

(1) $$v_{i}=\frac{\sum_{j=1}^{m}{\bar{v}}_{j}}{m}$$

Step 2: Divide the traffic network using a small grid, whose size is (0.0001° × 0.0001°, longitude and latitude), where 0.0001 in longitude (or latitude) in Beijing is equal to approximately 10 m (shown in Figure 3b), and each grid box represents a spatial region.

Step 3: Map the average speed to the grid. The value of a blank area is set to zero; if multiple links pass through the same grid box, we assign their average speed to the box (as shown in Figure 3c) and scale the speed to (0, 1) (as shown in Figure 2c).

Using the grid-based network-segmentation method, the relative topology among different links remains unchanged. This treatment can retain the geometric information of roads, such as sharp U-turns and interchanges at a fine granularity.

### 3.2. Spatial Features Captured by a CNN

**Figure 4:**
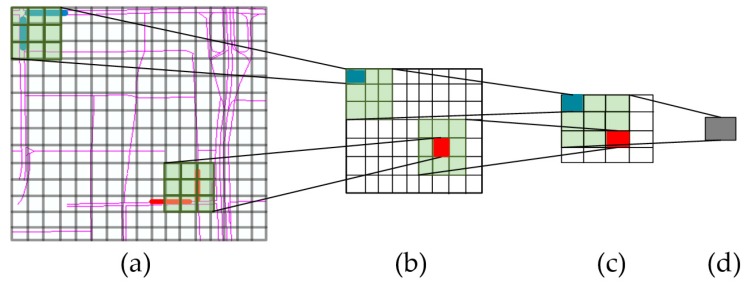
Convolutions for capturing near and far dependencies: (**a**) the original transportation network; (**b**) the transformed network with the first convolution; (**c**) the transformed network with the second convolution; (**d**) the transformed network with the third convolution.

The congestion in one link not only affects its most adjacent links, but may also propagate to other far-side regions. CNNs have been successful in extracting features. In this study, we construct deep convolutional neural networks (DCNNs) to capture the spatial relationships among links. The spatial dependencies of nearby links (the lines with the same colors in Figure 4a) can be mined by the shallow convolutional layer and the spatial dependencies for more distant links (the lines with different colors in Figure 4a) can be extracted by the deep convolutional layer, because the distance among them will be shortened due to the convolution and pooling processes. For example, each grid box represents a spatial region (similar to the network representation in step 2) in Figure 4a, the transparent green region represents a 3 × 3 convolutional filter, the lines with the same colors represent two nearby links, and the lines with different colors represent two distant links. With the convolution and pooling procedure in CNN, the distance between the blue line and red line in Figure 4b becomes shorter than that in Figure 4a. These abstract features are significant for the prediction problem [45].

**Figure 5:**
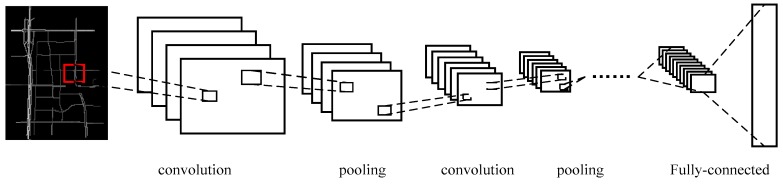
Structure of the DCNNs.

We naturally utilize a 2D CNN to capture the spatial features of the traffic network. The input for DCNNs is an image (Figure 2c) that represents one traffic state, and the pixel values in the image range from 0 to 1. The network framework is shown as Figure 5, including the input layer, convolution layer, pooling layer, fully connected layer, and output layer. The details of each part are subsequently explained.

The input image at time t to the DCNNs is set to $A^{T}=\left\{a_{m,n}^{t}\right\}$, where m and n represent the latitude coordinates and longitude coordinates, respectively, and the output of the DCNNs at time t is set to $X^{t}={\left\{x_{u}^{t}\right\}}_{u=1}^{p}$, where p is the number of links in the traffic network. The feature extraction is performed by convolving the input with filters. Denote the r-th filter output of the l-th layer as $O_{r}^{l}$, and denote the k-th filter output of the previous layer as $O_{k}^{l-1}$. Thus, $O_{r}^{l}$ can be calculated by Equation (2), where $W_{kr}^{l}$ and $b_{r}^{l}$ are the weight and the bias, ∗ denotes the convolution operation, and f is a nonlinear activation function. After convolution, max-pooling is employed to select the salient features from the receptive region and to greatly reduce the number of model parameters by merging groups of neurons.

(2) $$O_{r}^{l}=f\left(\sum_{k}W_{kr}^{l}*O_{k}^{l-1}+b_{k}^{l}\right)$$

### 3.3. Long Short-Term Temporal Features

Traffic data has a distinct temporal dependency, such as video and language, and the traffic state several hours earlier may have a long-term impact on the current state. The most successful model for handling long-term time series prediction is LSTM, which achieves a powerful learning ability by enforcing constant error flow through designed special units [10]. Traditional RNNs suffer from vanishing or exploding gradients when the number of time steps is large. LSTMs introduce memory units to learn whether to forget previous hidden states and update hidden states; they have been shown to be more effective than traditional RNNs [46]. Motivated by the temporal dynamics of traffic flow and the superior performance in long-term time-series prediction, we explore the application of LSTMs as a key component in predicting spatiotemporal traffic speeds in a large-scale transportation network.

**Figure 6:**
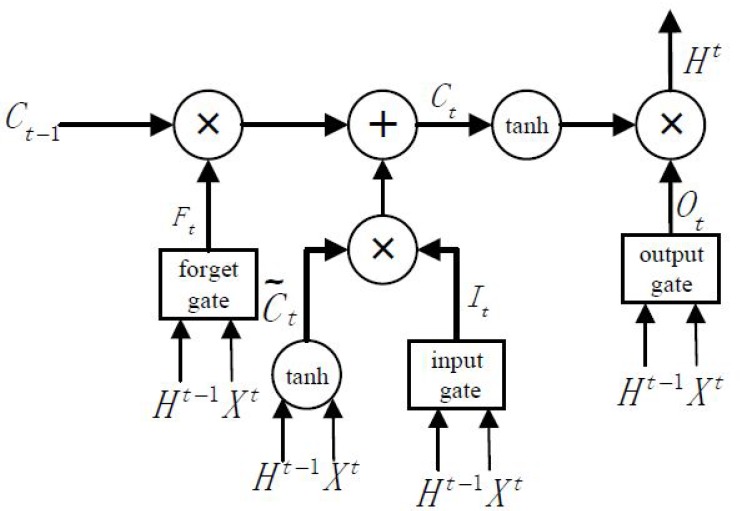
LSTM NN architecture.

LSTMs are considered to be a specific form of RNNs; each LSTM is composed of one input layer, one or several hidden layers, and one output layer. The key to LSTMs is a memory cell, which is employed to overcome the vanishing and exploding gradients in traditional RNNs. As shown in Figure 6, the LSTMs contains three gates, namely, the input gate, forget gate, and output gate. These gates are used to decide whether to remove or add information to a cell state.

In this paper, at time t, the output $X^{t}={\left\{x_{u}^{t}\right\}}_{u=1}^{p}$ of DCNNs represents the input of LSTMs, and the output of LSTMs is denoted as $H_{t}={\left\{h_{u}^{t}\right\}}_{u=1}^{q}$, where q represents the number of hidden units. The cell input state is ${\tilde{C}}_{t}$ , the cell output state is $C_{t}$, and the three gates’ states are $I_{t},F_{t},O_{t}$ . The temporal features of the traffic state will be iteratively calculated according to Equations (3)–(8):

(3) $$\mathrm{Input}\;\mathrm{gate}:I_{t}=\sigma \left(W_{1}^{i}X_{q}+W_{h}^{i}H_{t-1}+b_{i}\right),$$

(4) $$\mathrm{Forget}\;\mathrm{gate}:\;F_{t}=\sigma \left(W_{1}^{f}X_{t}+W_{h}^{f}H_{t-1}+b_{f}\right),$$

(5) $$\mathrm{Output}\;\mathrm{gate}:\;O_{t}=\sigma \left(W_{1}^{o}X_{t}+W_{h}^{o}H_{t-1}+b_{o}\right),$$

(6) $$\mathrm{Cell}\;\mathrm{input}:\;{\tilde{C}}_{t}=\tanh\left(W_{1}^{c}X_{t}+W_{h}^{c}H_{t-1}+b_{c}\right),$$

(7) $$\mathrm{Cell}\;\mathrm{output}:\;C_{t}=I_{t}⊙{\tilde{C}}_{t}+F_{t}⊙C_{t-1},$$

(8) $$\mathrm{Hidden}\;\mathrm{layer}\;\mathrm{output}:\;H_{t}=O_{t}⊙\tanh\left(C_{t}\right),$$

where $W_{1}^{i},W_{1}^{f},W_{1}^{o},W_{1}^{c}$ are the weight matrices that connect $X^{t}$ to the three gates and the cell input; $W_{h}^{i},W_{h}^{f},W_{h}^{o},W_{h}^{c}$ are the weight matrices that connect $H^{t-1}$ to the three gates and the cell input; $b_{i},b_{f},b_{o},b_{c}$ are the biases of the three gates and the cell input; σ represents the sigmoid function; tanh represents the hyperbolic tangent function; and ⊙ represents the scalar product of two vectors.

### 3.4. Spatiotemporal Recurrent Convolutional Networks

The hypothesis made in this paper is that the spatiotemporal features of the traffic state can be learned by CNNs and LSTMs. The next step is to forecast the future traffic state by the integration of CNNs and LSTMs. The output of LSTMs is utilized as an input to a fully connected layer. The predicted speed value is calculated by Equation (9), where $W_{2},b$ represent the weight and the bias between the hidden layer and the fully connected layer, respectively, which demonstrates the output of the entire model, and the prediction vector $Y^{t+1}$ is of the same size as the number of links; and we train the model from end to end.

(9) $$Y^{t+1}=W_{2}\times H_{t}+b_{2}$$

**Figure 7:**
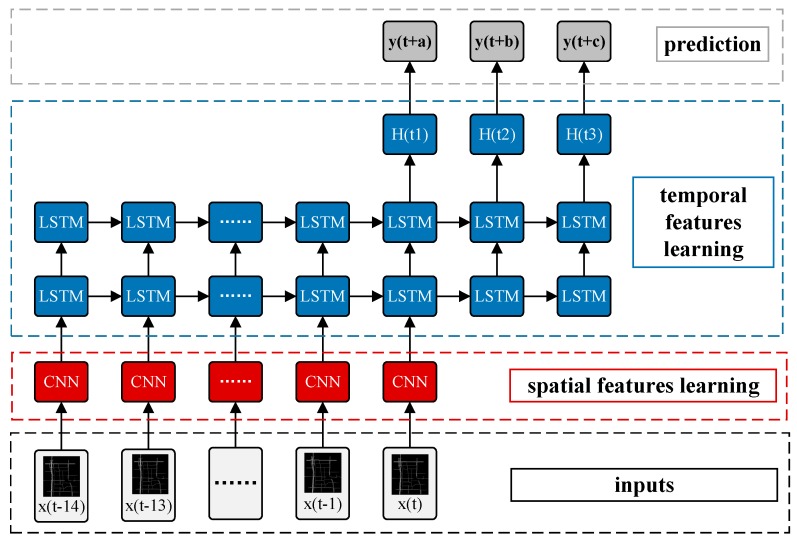
Framework of SRCNs.

In this section, we propose a novel deep architecture named a spatiotemporal recurrent convolutional network (SRCN) to predict the network-wide traffic state. A graphical illustration of the proposed model is shown in Figure 7. Each SRCN consists of a DCNN, two LSTMs, and a fully connected layer. The detailed structure of the SRCNs is described in the experiment section. The values of *a*, *b*, *c*, in Figure 7 can be arbitrarily established, which indicates that we can make multi-step predictions; for example, if we set *a*, *b*, *c*, to (2, 4, 5), we can predict the traffic states of the next (2, 4, 5) time steps based on the historical data of several steps.

## 4. Empirical Study

### 4.1. Data Source

The data used in this study originate from the floating cars with GPS devices in Beijing, and are transmitted to the traffic management center every two minutes. The key information for each record includes the longitude, latitude, timestamp, direction, and vehicle speed, etc. The duration of data collection is from 1 June 2015 to 31 August 2015 (92 days). All erroneous and missing data are properly remedied. The time period in this study ranges from 06:00:00 to 22:00:00, when high travel demand is commonly observed. Thus, 481 traffic states exist per day. The traffic network we tested is located between the Second Ring Road and Third Ring Road in Beijing. The network encompasses 278 links, and the total length of the network exceeds 38.486 km, including seven arterial roads and hundreds of interchanges and intersections. The data were divided into two subsets: data from the first two months were employed for training, and the remaining data were employed for testing, and thus the number of training and testing samples is 29,340 and 14,910, respectively.

For all methods, the time lag is set to 15, which indicates that the traffic states of the previous 15 × 2=30 min were used to predict future traffic states. For example, if a=15 (See in Figure 7), historical data from the previous 30 min are used to predict the traffic state 30 min in the future. Different settings are tested in the following experiments.

### 4.2. Implementation

The details of our SRCNs are shown in Table 1. SRCNs are trained based on the optimizer RMSprop [47], which has been proven to work well [48], especially in the RNN model [49]. The learning rate is set to 0.003; the decay parameter is set to 0.9; and the batch size is set to 64. The loss function is the mean squared error (MSE) and the validation-data proportion is set to 20%. A batch-normalization layer is used to overcome internal covariate shift. Since our model is very deep, we can also employ a substantially higher learning rate to accelerate convergence [50]. The dropout layer and early stopping are used to prevent overfitting [51], and all parameters of our model are dependent on numerous experiments to yield an optimal structure and the normal distribution N(0,1) is utilized to initialize the parameters. The SRCNs are built and implemented upon the Keras framework [52] and a Graphics Processing Unit (GPU) is used to accelerate the model learning procedure. The structures of other methods (LSTMs and SAEs) are established according to their papers.

According to paper [36], the LSTM model consists of one input layer, one LSTM layer, and one output layer; there are ten hidden neurons in the LSTM layer. According to paper [33], the SAE model is composed of one input layer, three hidden layers, and one output layer, and the number of hidden units in each hidden layer is (400, 400, 400).

### 4.3. Comparison and Analysis of Results

In this section, we employ traffic speed data from Beijing, China, to evaluate our model—SRCNs—and compare them with other deep NNs, including LSTMs [36], SAEs [35], DCNNs, and SVM. For the DCNN model, the structure is the same as the first part of the SRCNs. For the SVM model, the kernel function is the radial basis function (RBF), and the trade-off parameter “c” and width parameter “g” are calibrated using five-fold cross validation. For comparison and analysis, we specify two different conditions: (a,b,c)=(1,2,3) for short-term prediction and (a,b,c)=(10,20,30) for long-term prediction. The mean absolute percentage error (MAPE) and root mean squared error (MSE) are utilized to measure the performance of the traffic state forecasting in this paper, which are defined in Equations (10) and (11), where $y_{it}$ and $z_{it}$ denote the predicted traffic speeds and actual traffic speeds, respectively, at time t at location i , where m is the total number of predictions, and $n_{p}=m\;\times \;n$. In the experiment, the value of n is 278, and the value of m is 14,896, which indicates that we tested 278 links and 14,896 traffic states.

(10) $$MAPE=\frac{1}{n_{p}}\sum_{i=1}^{n}\sum_{t=1}^{m}\left(\frac{y_{it}-z_{it}}{y_{it}}\right)$$

(11) $$RMSE=\sqrt{\frac{1}{n_{p}}\sum_{i=1}^{n}{\sum_{t=1}^{m}\left(y_{it}-z_{it}\right)}^{2}}$$

### 4.4. Short-Term Prediction

**Figure 8:**
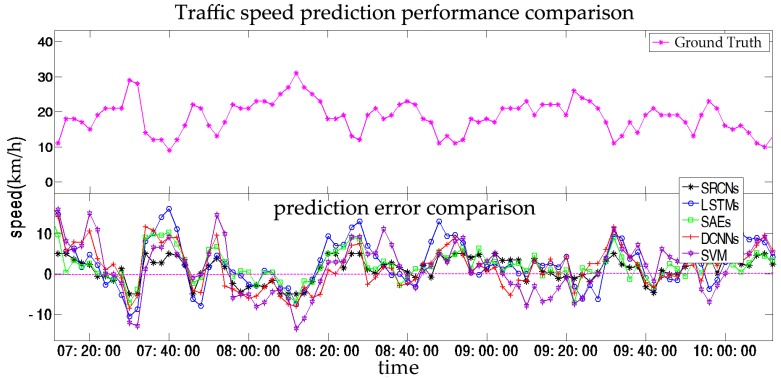
Traffic speed prediction performance comparison at 2 min time steps.

Short-term prediction is primarily employed for en-route trip planning and is desired by travelers who resort to in-vehicle navigation devices. In this section, we set (a,b,c)=(1,2,3), which indicates that we will predict traffic speeds in the next (2, 4, 6) min based on historical data from the previous 30 min. The results of the SRCNs, LSTMs, SAEs, DCNNs, and SVM are listed in Figure 8 and Table 2.

**Figure 9:**
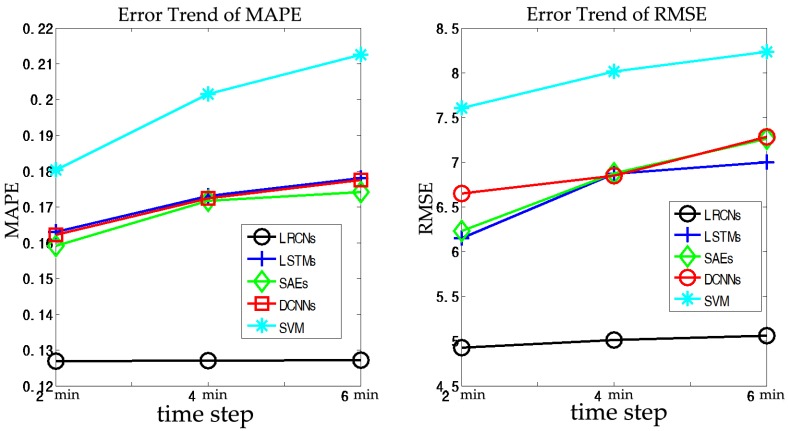
Prediction errors for the prediction horizons of (2, 4, and 6) min in terms of the MAPE and RMSE.

In this section, we compare SRCNs with four other algorithms (LSTMs, SAEs, DCNNs, and SVM) in terms of short term prediction. As shown as Figure 8, the upper plot is the ground truth and the lower plot presents the error deviated from the ground truth from the five different algorithms. It can be found that the SRCNs are the closest to the base line, while SVM fluctuates the most significantly. We observe that SRCNs yield the most accurate results for short-term traffic speed prediction in terms of MAPE and RMSE; the results are shown as Figure 9. One possible reason for this is that SRCNs consider spatiotemporal features. In the order listed in Table 2, the average MAPE values for the other algorithms decrease by 34.96%, 32.60%, 34.41%, and 56.22%, and the average RMSE values for the other algorithms decrease by 35.06%, 35.78%, 38.59%, and 59.02%, respectively. The SVM model exhibits the worst prediction performance, and the LSTMs and DCNNs show a similar precision, which indicates that spatial and temporal features have similar roles in short-term traffic prediction. The MAPE of SRCNs is approximately 0.1, and the RMSE is approximately 5. As shown in Figure 9, we observe that the prediction error increases as the prediction horizon increases. SRCNs yield the lowest prediction error with a stable trend.

### 4.5. Long-Term Prediction

**Figure 10:**
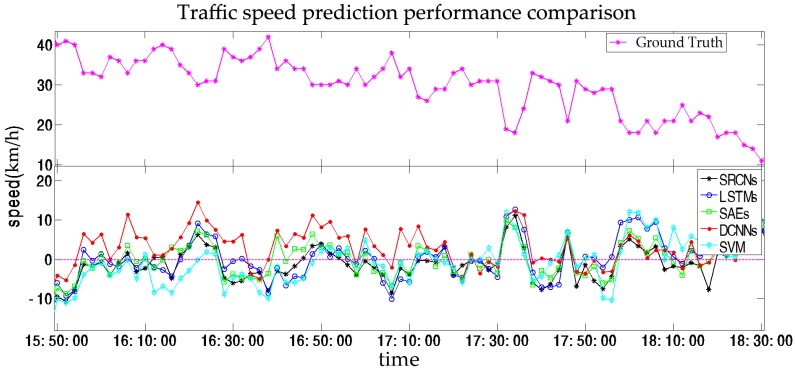
Traffic speed prediction performance comparison at a 20 min prediction horizon.

Long-term prediction, which is primarily adopted by pre-route travelers who plan their trips in advance, is considered to be more challenging than short-term prediction. In this section, we set (a,b,c)=(10,20,30), which indicates that we will predict the traffic speed in the next (20, 40, 60) min based on historical data for the previous 30 min. The results of the SRCNs, LSTMs, SAEs, DCNNs, and SVM are listed in Figure 10 and Table 3.

**Figure 11:**
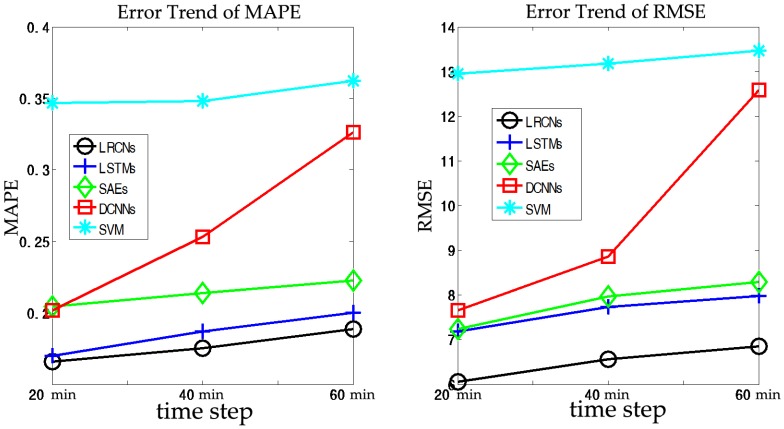
Prediction errors for the prediction horizons (20, 40, and 60) min in terms of MAPE and RMSE.

In this section, we compare SRCNs with four algorithms (LSTMs, SAEs, DCNNs, and SVM) in terms of long term prediction. From Figure 10, we can discover that the errors of five different algorithms for long term prediction deviate from the “zero line” more seriously than those for short term prediction. However, we observe that the SRCN model is still superior to other models in long-term traffic speed prediction in terms of MAPE and RMSE, as shown in Figure 11. This finding confirms the advantages of SRCNs in utilizing the spatiotemporal features in traffic networks. The average MAPE values for the other algorithms, in the order listed in Table 3, decrease by nearly 18.58%, 20.87%, 47.29%, and 100.34% relative to the SRCNs in long-term prediction. The average RMSE values for the other algorithms decrease by 17.62%, 20.69%, 49.45%, and 103.41%, respectively. Similar to short-term prediction, the SVM model exhibits the worst prediction performance. However, LSTMs perform much better than DCNNs, which indicates that spatial information contributes more than temporal features for long-term traffic prediction. The SRCNs outperform other algorithms, with the lowest MAPE—approximately 0.2—and an RMSE of approximately 6. As shown in Figure 11, we discover that the error increases as the prediction horizon increases, but the long-term prediction performance decays more rapidly than the short-term prediction performance.

SRCNs achieve the best accuracy compared with the other four algorithms (LSTMs, SAEs, DCNNs, and SVM) in both short- and long-term traffic speed prediction and obtain the most stable error trend, because SRCNs can learn both spatial and temporal features on a network-wide scale. SRCNs can perform multi-step-ahead prediction due to the special structure of LSTMs and can output a sequence of predictions [53]. These results verify the superiority and feasibility of the SRCNs, which employ deep CNNs to capture the special features and mine temporal regularity using an LSTM NN.

## 5. Conclusions

Inspired by the research findings of motion prediction in the domain of computer vision, where the future movement of an object can be estimated from a sequence of scenes generated by the same object, we proposed a novel grid-based transportation network segmentation method. The network-wide traffic can be snapshot as a series of static images and can retain the complicated road network topology, including interchanges, intersections, and ramps. Based on the proposed network representation method, a novel deep learning architecture named SRCN is developed; this method inherits the advantages of both DCNNs and LSTMs. DCNNs are employed to capture the near- and far-side spatial dependencies among different links, and LSTMs are utilized to learn the long-term temporal dependency of each link. To validate the effectiveness of the proposed SRCNs, traffic speed data were collected for three months with an updating frequency of 2 min from a Beijing transportation network with 278 links. Data from the first two months were employed for training, and the remaining data were employed for testing. In addition, three prevailing deep learning NNs (i.e., LSTMs, DCNNs, and SAEs) and a classical machine learning method (SVM) were compared with the SRCNs for the same dataset. The numerical experiments demonstrate that the SRCNs outperformed other algorithms in terms of accuracy and stability, which indicates the potential of combining DCNNs with LSTMs for large-scale network-wide traffic prediction applications.

The train-test process is very time consuming, and requires 19.4 h to complete the algorithm execution for a single parameter setting, even with GPU acceleration. Therefore, performing an N-fold cross validation is not realistic. A possible countermeasure is to deploy distributed or cloud computing based GPU-optimized servers for cross-validation.

In future studies, the model can be improved by considering additional factors, such as the weather, social events, and traffic control. In order to test the robustness of the proposed model, more data in different cities are required to validate the seasonal variation effect on the prediction accuracy. Moreover, the training efficiency can be also enhanced by optimizing pre-training methods, which may reduce the number of iterations while achieving more accurate results. Another intriguing research direction is to develop novel transportation network representation approaches. By eliminating the blank regions without any roadway network, the computational burden of training SRCNs should be greatly reduced. In addition, we aim to expand the transportation network to a larger scale.

## Figures and Tables

**Table 1:** Parameter settings of SRCNs.

Layer	Name	Channels	Size
0	Inputs	1	(163,148)
1	Convolution	16	(3,3)
	Max-pooling	16	(2,2)
	Activation (relu)	——	——
	Batch-normalization	——	——
2	Convolution	32	(3,3)
	Max-pooling	32	(2,2)
	Activation (relu)	——	——
	Batch-normalization	——	——
3	Convolution	64	(3,3)
	Activation (relu)	——	——
	Batch-normalization	——	——
4	Convolution	64	(3,3)
	Activation (relu)	——	——
	Batch-normalization	——	——
5	Convolution	128	(3,3)
	Max-pooling	128	(2,2)
	Activation (relu)	——	——
	Batch-normalization	——	——
6	Flatten	——	——
7	Fully connected	——	278
8	Lstm1	——	800
	Activation (tanh)	——	
9	Lstm2	——	800
	Activation (tanh)	——	——
10	Dropout (0.2)	——	——
11	Fully connected	——	278

**Table 2:** Comparison of different methods in terms of short-term prediction.

	Time Steps	2 min		4 min		6 min		Average Error	
Algorithm		MAPE	RMSE	MAPE	RMSE	MAPE	RMSE	MAPE	RMSE
SRCNs		0.1269	4.9258	0.1271	5.0124	0.1272	5.0612	0.1270	4.9998
LSTMs		0.1630	6.1521	0.1731	6.8721	0.1781	7.0016	0.1714	6.7527
SAEs		0.1591	6.2319	0.1718	6.8737	0.1742	7.2602	0.1684	6.7886
DCNNs		0.1622	6.6509	0.1724	6.8516	0.1775	7.2845	0.1707	6.9290
SVM		0.1803	7.6036	0.2016	8.0132	0.2123	8.2346	0.1984	7.9505

**Table 3:** Comparison of different methods in terms of long-term prediction.

	Time Steps	20 min		40 min		60 min		Average Error	
Algorithm		MAPE	RMSE	MAPE	RMSE	MAPE	RMSE	MAPE	RMSE
SRCNs		0.1661	6.0569	0.1753	6.5631	0.1889	6.8516	0.1768	6.4905
LSTMs		0.1700	7.1857	0.1872	7.7322	0.2003	7.9843	0.1858	7.6340
SAEs		0.2045	7.2374	0.2139	7.9737	0.2228	8.2881	0.2137	7.8331
DCNNs		0.2018	7.6608	0.2531	8.8613	0.3264	12.5846	0.2604	9.7022
SVM		0.3469	12.9577	0.3480	13.181	0.3621	13.4676	0.3542	13.2021
